# Supplementary material for: A Novel Cold-Adapted and High-Alkaline Alginate Lyase with Potential for Alginate Oligosaccharides Preparation
Source: Molecules. 2023 Aug 22;28(17):6190. doi: 10.3390/molecules28176190 (PMC10488352; doi:10.3390/molecules28176190)
Supplement: Supplementary file 1 [file molecules-28-06190-s001.zip › molecules-2511811-supplementary.pdf]

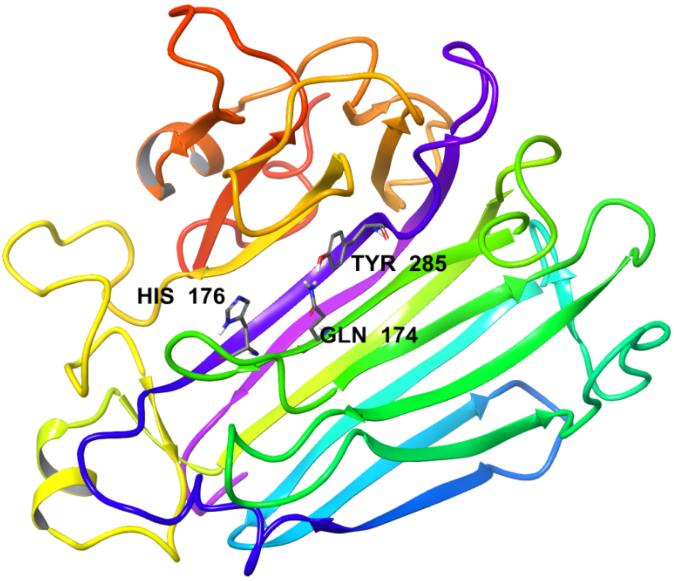

Figure S1. 3D structure of Alyw208 by homology modeling. Three predicted catalytic residues (Q174, H176, and Y285) are shown.

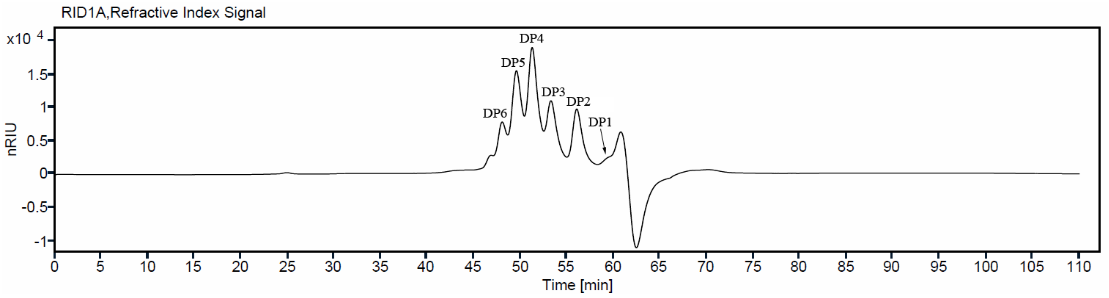

Figure S2. SEC analysis of the degradation products. Alginate monomer (DP1) and AOs with DPs 2-6 were the main products derived from enzymatic degradation of 2% sodium alginate by recombinant alginate lyase Alyw208.

Table S1. The proportions of AOs with different DPs in the final degradation product.

DP	Retention time (min)	Mass fraction (%)
2	56.053	16.47
3	53.280	18.11
4	51.266	28.62
5	49.577	21.91
6	48.050	9.75
>6	46.866	5.14
